# Mapping the Magnetic Coupling of Self-Assembled Fe_3_O_4_ Nanocubes by Electron Holography

**DOI:** 10.3390/ma14040774

**Published:** 2021-02-06

**Authors:** Lluís López-Conesa, Carlos Martínez-Boubeta, David Serantes, Sonia Estradé, Francesca Peiró

**Affiliations:** 1 Laboratory of Electron Nanoscopies (LENS-MIND), Departament d’Enginyeria Electrònica i Biomèdica, Universitat de Barcelona, 08028 Barcelona, Spain; sestrade@ub.edu (S.E.); francesca.peiro@ub.edu (F.P.); 2 Institute of Nanoscience and Nanotechnology, Universtitat de Barcelona, (IN2UB), 08028 Barcelona, Spain; 3 Centres Científics i Tecnològics de la Universitat de Barcelona (CCiTUB), 08028 Barcelona, Spain; 4 Freelancer in O Con, 36950 Moaña, Spain; cboubeta@gmail.com; 5 Ecoresources P.C., 54627 Thessaloniki, Greece; 6 Instituto de Investigacións Tecnolóxicas and Departamento de Física Aplicada, Universidade de Santiago de Compostela, 15782 Santiago de Compostela, Spain; david.serantes@usc.es

**Keywords:** electron holography, magnetic nanoparticles, magnetic hyperthermia

## Abstract

The nanoscale magnetic configuration of self-assembled groups of magnetite 40 nm cubic nanoparticles has been investigated by means of electron holography in the transmission electron microscope (TEM). The arrangement of the cubes in the form of chains driven by the alignment of their dipoles of single nanocubes is assessed by the measured in-plane magnetic induction maps, in good agreement with theoretical calculations.

## 1. Introduction

Magnetic hyperthermia has been the subject of intense research in recent years. Among the potential applications, it allows for a complementary approach to standard therapies for cancer treatment (for review, see e.g., [1]). This technique offers the advantage of delivering a highly localized damage via the targeting of tumor cells with magnetic nanoparticles. By exciting these nanoparticles with a radio-frequency signal, local heating of the surrounding area is achieved, with lower full-system toxicity than chemotherapy and without ionizing radiation affecting healthy tissue, as in the case of radiotherapy. However, in spite of having shown some promising results on palliative care, the high particle concentration required rises concerns about the toxicity and side effects of the treatment. Thus, improving efficiency by optimizing the magnetic response of nanoparticles is crucial in order to obtain therapeutic effects while keeping the number of nanoparticles as low as possible.

In this regard, performance is governed mainly by size distribution, saturation magnetization (M_S_), and magnetic anisotropy (K) [2,3]. For a given excitation AC amplitude and frequency, these three are the parameters to tune in order to optimize the inductive specific absorption rate (SAR) of the system, usually reported in watts per gram [4]. To date, the highest reported SAR values correspond to metallic Fe nanocubes [5]. However, the low chemical stability of metallic nanoparticles under physiological conditions make the magnetically softer magnetite (Fe_3_O_4_) a much more promising candidate for applications in magnetic hyperthermia [6]. On the one hand, selecting Fe_3_O_4_ as the material of choice fixes a value for M_S_. On the other hand, the particular application limits the range of particle sizes between the superparamagnetic limit (≥15 nm) and the optimal size for internalization into mammalian cells (≤50 nm) [7,8]. Thus, the remaining free parameters in order to optimize the heating response of the nanoparticles are the magnetic anisotropy (K) [9] and the volume fraction [4].

A way to increase magnetic anisotropy is by properly tuning the shape of the particles. Taking into account that a sphere has the minimum surface to volume ratio, cubic nanoparticles are already an improvement when compared to spherical ones because of their higher surface magnetic anisotropy. Another contribution to a larger surface anisotropy is the presence of well-defined atomic planes at the surfaces [10]: this is also in favor of the cubic shape, considering the most irregular crystal facets corresponding to a spherical nanoparticle.

An additional consequence of the cubic shape is an increased tendency of the magnetic nanoparticles to arrange in chains by sharing flat surfaces. The formation of ensembles of nanoparticles is also a way of engineering the magnetic response via the modification of the strength of the dipolar interaction between nanoparticles. Theoretical calculations for the hysteresis loops considering chains of Fe_3_O_4_ for different numbers of dipole-aligned nanocubes are reported in Boubeta et al. [11]. The simulations show an increasing area of the loop when increasing the number of aligned particles, therefore resulting in a potentiation of the heating efficiency. Furthermore, the thermal stability gained by creating arrays, also shown by simulations of magnetic response versus temperature, is an advantage when exploiting hysteresis losses. These results indicate a promising way to increase the hyperthermia performance by assembling cubic particles in elongated chains. On the heels of our previous article, here we use electron holography experiments to access and map the magnetic configuration of Fe_3_O_4_ cubic nanoparticles whose average diameter of 40 nm is expected to be close to the 180° domain wall width [12], thus may be promoting the presence of vortex pseudo-single-domain configurations [13,14].

## 2. Materials and Methods

Magnetite nanocube synthesis was performed following the one-pot and two-step procedure described previously [11]. Shortly, this requires the thermal decomposition of Fe(acac)_3_ in boiling dibenzylether under argon atmosphere in the presence of decanoic acid. After cooling down, acetone was added to yield a precipitate, which was then separated by centrifugation. The supernatant was discarded and the particles were redispersed in chloroform. Samples for transmission electron microscopy (TEM) observation were prepared by dispersing a drop of the nanoparticle solution on a carbon-coated copper grid.

High resolution HRTEM experiments were carried out in a JEOL J2100 (Tokyo, Japan) located at CCiTUB. Electron holography experiments were carried out in the Hitachi I2TEM microscope (Tokyo, Japan) at CEMES-CNRS in Toulouse. The I2TEM is a modified Hitachi HF3300C TEM equipped with a 300 kV cold FEG, with an aberration corrector in the objective system and a 4k × 4k CCD camera. The I2TEM has an additional specimen holder port placed above the objective lens so that its magnetic field does not affect the specimen during the whole experiment. In this configuration, the aberration-corrected objective lens can be used as a Lorenz lens.

Micromagnetic simulations were performed with the OOMMF software package (version 1.0) [15], under the assumption that the nanocubes are perfectly cubic and identical. Each particle was discretized in 3D cells of 2 nm side, with a nonmagnetic intercube separation of 2 nm. We used bulk magnetic parameters for magnetite: M_S_ = 477 kA/m, cubic magnetocrystalline anisotropy K = −11 kJ/m^3^, and exchange coupling constant of 1.0 × 10^−11^ J/m. The simulation procedure was to saturate the chains and let them relax to equilibrium at T = 0.

## 3. Results and Discussion

Our earlier studies [11] revealed a generalized self-assembly of Fe_3_O_4_ nanocubes in chain-like structures. Nanocubes are rather homogeneous in size, with ∼40 nm lateral dimension. There was no apparent contrast variation within each nanoparticle, thus suggesting that particles were completely oxidized during synthesis. The magnetic properties of the particles are compatible with Fe_3_O_4_, with an incontrovertible evidence of Verwey transition around 120 K. HRTEM images confirmed monocrystalline Fe_3_O_4_ nanocubes indexed according to the inverse spinel structure of iron oxide.

Electron tomography [11] was used to reconstruct the 3D volume of a Fe_3_O_4_ nanoparticle chain. Results allowed accessing the shape of the chain in 3D and, at the same time, segmentation of the information down to single particle level. The cubic shape was confirmed by the 3D reconstruction, as well as cube alignment by sharing {100}-type flat faces. A separation in the order of ∼2 nm was found between adjacent cubes, corresponding to the organic ligand chains. At this surfactant layer thickness, van der Waals interaction between adjacent cubes is expected to be low [16], so the self-assembly could be ascribed to the magnetic dipole-dipole interaction.

Structural and morphological TEM characterization at the nanoscale, as well as macroscopic magnetic measurements, are in good agreement with the proposed model and the corresponding simulation reported previously [11]. However, this constitutes an indirect evidence of the magnetic coupling of the nanostructures. Direct evidence, namely real space imaging of the magnetic ordering down to single particle level, can be provided by electron holography [17,18,19].

**Figure 1:**
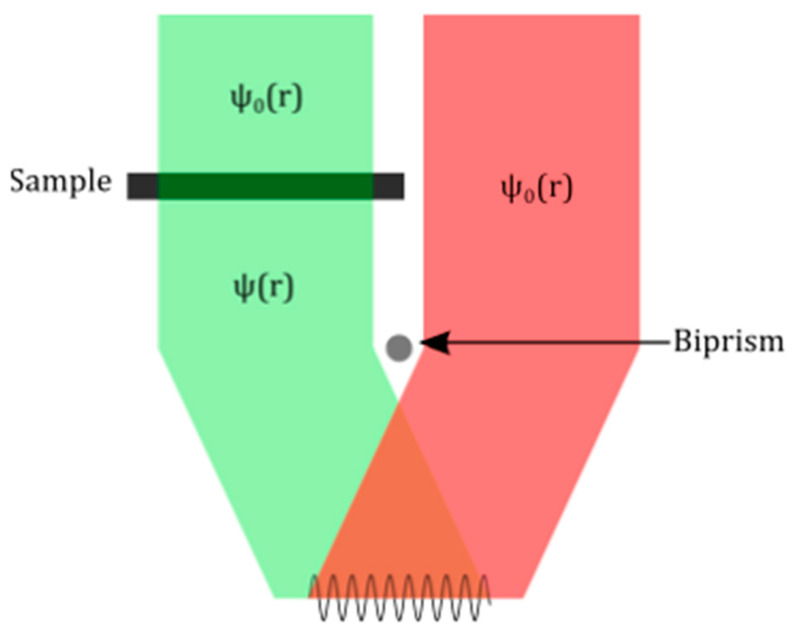
Off axis electron holography basic diagram. The electron wave resulting from the interaction of the electron beam with the sample, and a reference electron wave from the electron beam travelling along the vacuum, are made to interfere using an electrostatic biprism. The resulting interference fringe pattern is studied.

In order to assess the magnetic state of the Fe_3_O_4_ ensembles, “up and down” electron holography experiments were carried out using two electrostatic biprisms. Which consists in acquiring two sets of holograms (sample and vacuum reference) corresponding to the two possible orientations of the TEM specimen. This requires taking the sample out of the microscope and flipping it between the two acquisitions. A hologram is formed by the superposition of two electron beams on the detector: one beam has travelled through the specimen and the other one has travelled through vacuum. The superposition of the two beams is obtained using an electrostatic biprism (in our setup, the lower one), as depicted in Figure 1. The resulting hologram contains interference fringes due to the phase shift caused by the specimen on the electron beam that travelled through it.

**Figure 2:**
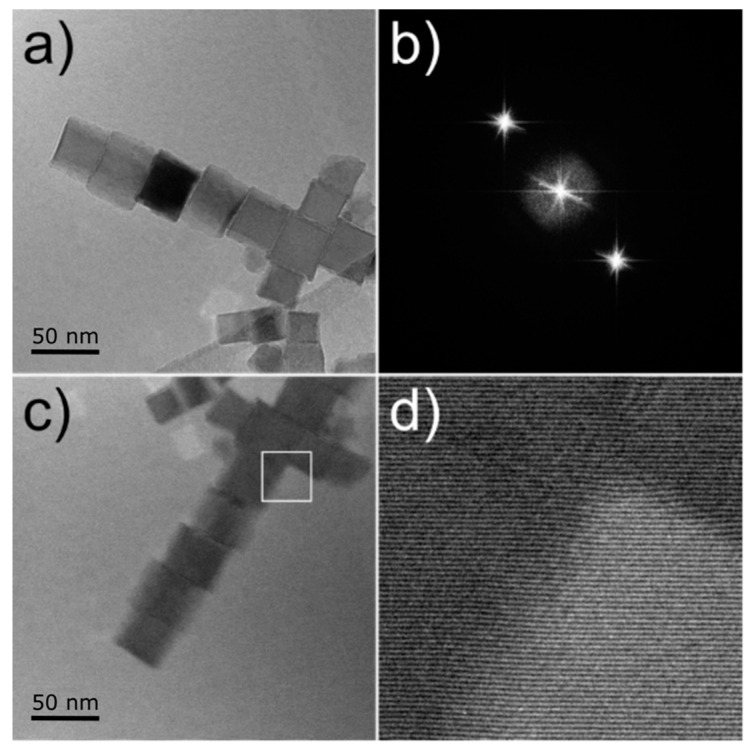
(**a**) Hologram covering a nanoparticle ensemble formed by two crossing chains. (**b**) FFT of the hologram showing the center band and sidebands. No spots corresponding to Fresnel fringes are visible due to the use of a two biprism configuration. (**c**) Hologram of the same ensemble obtained after mechanical flipping of the sample. (**d**) Detail of the interference fringes from the region highlighted in (**c**).

Figure 2a,c show the two flip-related holograms for an ensemble of nanocubes. The use of two electrostatic biprisms allows decoupling two important parameters: the width of the superposition region and the interference fringes spacing [20]. When working in a single biprism configuration, the applied voltage defines both parameters, so that a balance needs to be found. The use of two biprisms allows controlling them separately by defining different voltages for each one of them. An additional advantage of this configuration is the elimination of Fresnel interference fringes in the holograms when the lower biprism is in the region shaded by the upper one. This can be seen in Figure 2b, where only the centerband and the sidebands are present in the Fourier transform. This results in a higher fringe contrast, which is a key parameter limiting the magnetic signal resolution. The obtained small fringe spacing and high contrast in the recorded holograms is illustrated in Figure 2d.

**Figure 3:**
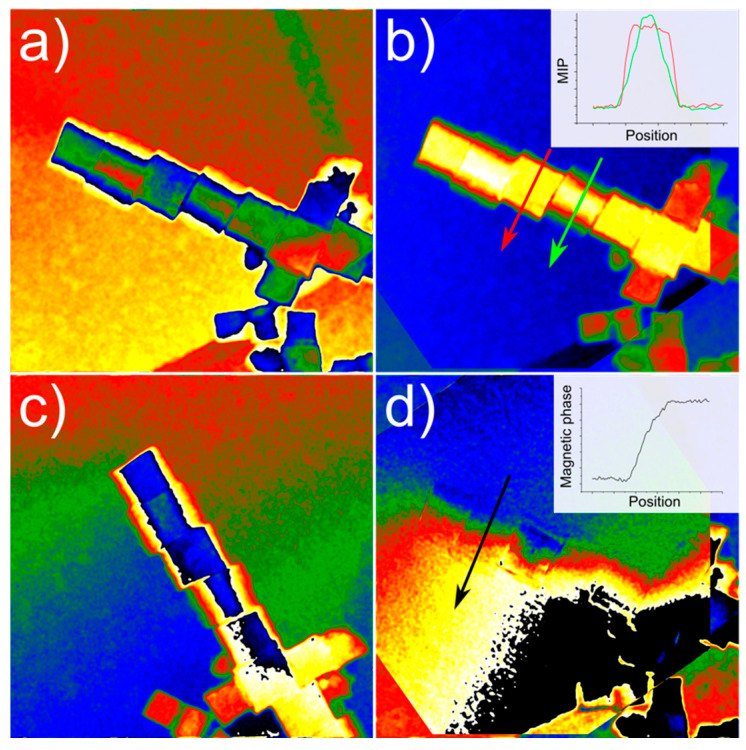
(**a**,**c**) Phase shift maps corresponding to the “up” and “down” holograms, respectively. The “down” image needs to be flipped so that it can be aligned with the “top” image. (**b**) Phase sum image, corresponding to the mean inner potential (MIP). (**d**) Phase difference image, corresponding to the magnetic phase shift. The magnetic phase difference across the object, in the direction of the black arrow and shown in the inset, indicates its magnetic nature.

After subtracting the constant phase term corresponding to the vacuum reference holograms for both the up and down configurations (not shown here), and correcting the images for the mechanical flip process, a mask is set on one of the sidebands, and the corresponding amplitude and phase are calculated. The obtained phase shift maps for the up and down holograms of the ensemble under study are shown in Figure 3a,c, respectively. Considering the experimental setup, the only actual contributions to the phase shift (*φ*) are the electrostatic and the magnetic phases. Each one of the phase maps will have contributions from both electrostatic ($\varphi_{E}$) and magnetic ($\varphi_{M}$) components

(1) $$\varphi_{up}=\;\varphi_{E,up}+\varphi_{M,up}$$

(2) $$\varphi_{down}=\;\varphi_{E,down}+\varphi_{M,down}$$

Given the flipping process between the two acquisitions and the nature of the electrostatic and magnetic fields, the phase shifts resulting from the two holograms will satisfy the following relationship

(3) $$\varphi_{E,up}=\varphi_{E,down}$$

(4) $$\varphi_{M,up}=-\varphi_{M,down}$$

So, after careful alignment of the phase shift maps, simple phase operations allow separating the magnetic phase

(5) $$\varphi_{M}=\;\frac{\varphi_{up}-\varphi_{down}}{2}$$

from the electrostatic phase corresponding to the mean inner potential (MIP) of the sample.

(6) $$\varphi_{E}=\;\frac{\varphi_{up}+\varphi_{down}}{2}$$

The resulting phase sum and difference maps are shown in Figure 3b,d, respectively. The dependence of the MIP is on the electric charge distribution and sample thickness so, considering a homogeneous material, an intensity profile across the sample can provide information on the third dimension. The MIP intensity profiles show sharper edges for the cube presenting a stronger diffraction contrast, as could be expected from a cube lying flat on one face and therefore closer to the zone axis. The phase difference map corresponding to the magnetic signal shows a phase shift with a frontier laying along the direction of the nanocube chain. This magnetic phase difference, clearly shown in the intensity profile in the inset, is a clear signature of the magnetic behavior of the nanocubes.

From the obtained magnetic phase $\varphi_{M}$, the magnetic induction map in the specimen plane (perpendicular to the beam direction, $B^{p}\left(x,y\right)$) can be calculated as its gradient

(7) $$\vec{\nabla }\varphi_{M}\left(x,y\right)=\frac{e}{\hbar }\;\left[B_{y}^{p}\left(x,y\right)-B_{x}^{p}\left(x,y\right)\right]$$

**Figure 4:**
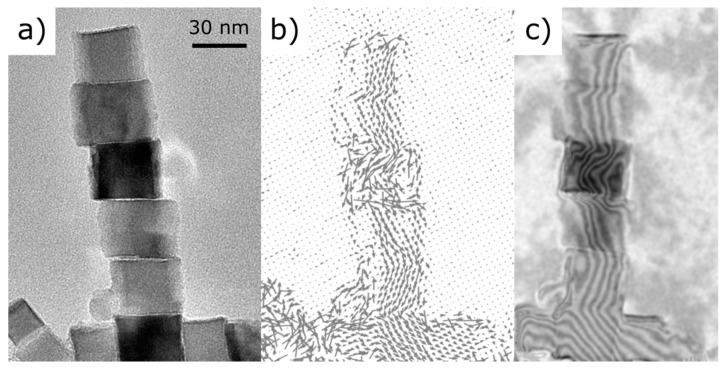
(**a**) Reference images. (**b**) In-plane induction map. (**c**) Magnetic phase signal visualized as a cos(nφ_M_) contour map superimposed to the amplitude image. Arrows and contours correspond to magnetic flux lines showing the magnetic coupling of the cubes. Stray field lines are visible at the tip of the chain.

A different way to visualize the magnetic coupling along the chain is by representing the magnetic phase shift as contour maps according to the expression $\cos(n\varphi_{M})$ for n = 1, 2, …. The resulting contours represent the change in magnetic phase and, thus, constitute a map of the in-plane magnetic flux lines. The induction vector map and contour map for the ensemble under study are shown in Figure 4b,c, next to the inverse fast Fourier transform (IFFT) of the hologram centerband as a geometrical reference (Figure 4a). The magnetic signal is somewhat distorted in the central nanocube showing a stronger diffraction contrast due to its crystal orientation, as mentioned before for the MIP map. Diffraction contrast decreases the interference fringes contrast, thus making the detection of the magnetic signal difficult. In this example, the ensemble is formed by two crossing chains: a long chain with N = 6 along the vertical direction and a shorter horizontal one with N = 3. Magnetic flux lines follow the alignment of the chains and rotate ∼55° in the “node” nanocube where the two chains intersect at a right angle.

**Figure 5:**
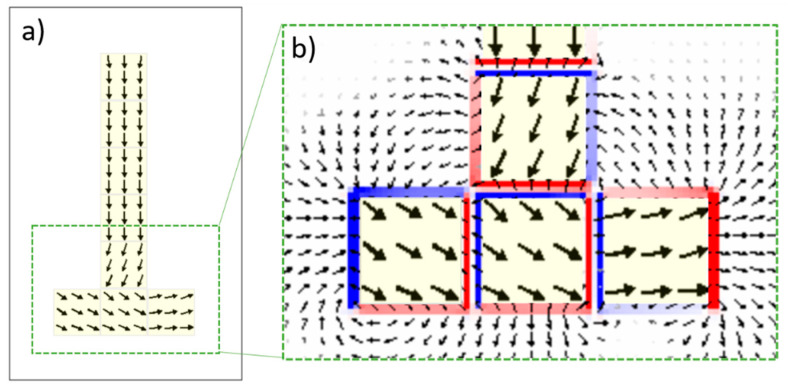
(**a**) Micromagnetic configuration at remanence of the magnetization of two crossing chains as those depicted in Figure 4. To ease the observation, each arrows stand for average magnetization over 7 × 7 × 7 unit cells. (**b**) Augmented view of the crossing-chains area, superimposed with the stray field configuration depicted by the small arrows external to the magnetic cubes (light yellow regions); for illustrative purposes the stray field arrows are averaged over 3 × 3 × 3 unit cells. The red-blue colors indicate the divergence of the stray fields.

One wonders whether such a peculiar magnetic configuration could be reproduced by micromagnetic calculations. In a naive picture, we can see the two chains depicted in Figure 4 as a T-shaped structure. As the sample has never been exposed to any magnetic field, the measured configurations should correspond to virgin remnant states. The results are shown in Figure 5 and correspond rather nicely to the experimental ones. On the one hand, the elongated structure introduces a uniaxial anisotropy of magnetostatic origin and defines the easy axis for the magnetization. On the other hand, the surfaces of the nanocubes correspond to [100] planes and as the magnetization attempts to flip between <111> easy crystallographic directions, the spins curling in the junction must have opposite helicities [17], and form an angle of θ = cos^−1^ (1/√3) ∼55°.

**Figure 6:**
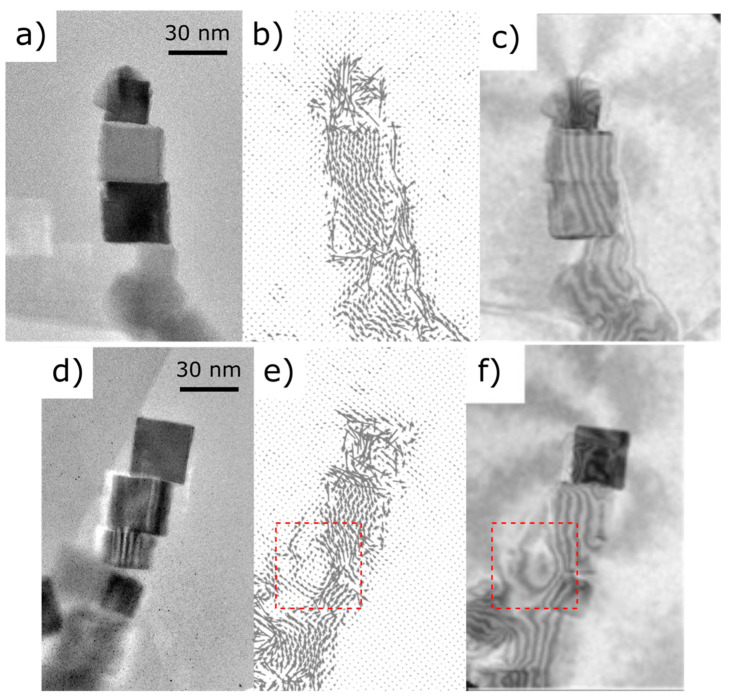
(**a**,**d**) Reference images. (**b**,**e**) In-plane induction maps. (**c**,**f**) Magnetic phase signal visualized as a cos(n*φ*_M_) contour map superimposed to the amplitude image. Arrows and contours correspond to magnetic flux lines showing the magnetic coupling of the cubes. Stray field lines are visible at the tip of the chains. A vortex spinning perpendicular to the chain axis is also visible in (**e**,**f**), highlighted by red squares. The two last crystals on the upper side of the chains show a complex magnetic state. Interactions between neighboring nanocubes induce a bending of the magnetic induction.

An analogous processing was carried out for holograms from different assemblies and the resulting induction vector maps and contour maps of the magnetic phase shift are shown in Figure 6. Both of them present a cooperative organization governed by the dipole–dipole interaction, despite their stronger spatial deviation from a perfectly aligned assembly. This is probably because they contain a bigger proportion of crystals of different sizes. Long reaching stray field lines are visible, particularly at the tips, both on simulated and experimental mappings, but close outside the field of view. Flux lines forming concentric circles can also be seen in Figure 6e,f. The contrast spot observed at the center of that nanocube corresponds to the turn out-of-plane magnetization [21], which leads to a drastic reduction of the dipolar energy.

**Figure 7:**
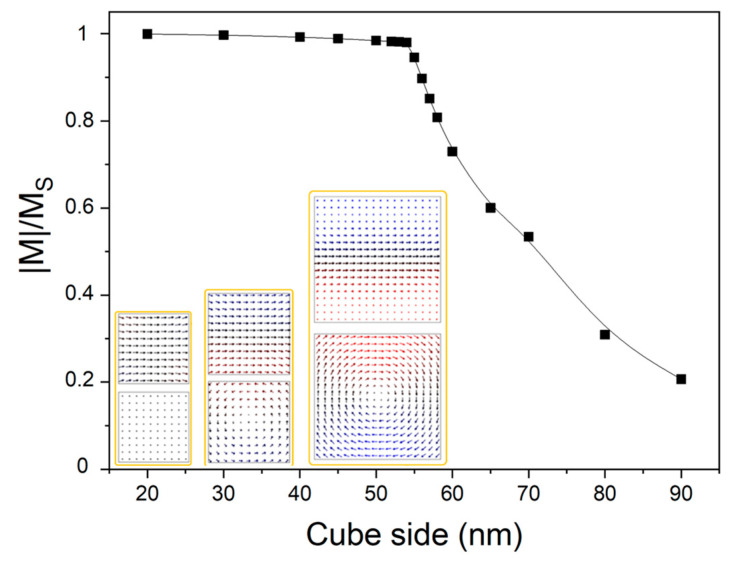
Size dependence of normalized magnetization. The snapshots show the remanent magnetization configurations, taken along two orthogonal directions, for exemplary dimensions (50, 58, and 90 nm). For clarity purposes the arrows representing the magnetization are average over 10 × 10 × 10 basic unit cells. All the particles are drawn at the same scale.

We will end by making at least a brief reference to such vortex configurations. Figure 7 shows the size dependence of the spontaneous magnetization of a cubic magnetite nanoparticle. With increasing particle sizes beyond ∼50 nm, the magnetization of a single domain vanishes indicating the 3D vortex flux closure structure. Additionally, for exploratory purposes we included (not-shown) an iron oxide outer layer of thickness 0–2 nm with the bulk maghemite magnetic parameters. This thin shell layer, however, does not seem to change the simulated magnetic configurations of the Fe_3_O_4_ nanocubes.

Similar calculations have been performed in the past, especially by Butler and Banerjee [12]. They found that stable single-domain cubic magnetite nanoparticles at 290 K exist in the transition region 40–76 nm imposed by the superparamagnetic limit and the cost of introducing domain walls. Accordingly, in another study Usov et al. [22] estimated it in about 56 nm in lateral size. Therefore, the overall agreement is reasonable considering the experimental errors and the zero temperature simulations. Moreover, a vortex-like state such the one depicted in Figure 6, which is now perpendicular to the chain axis, may also depend sensitively on the particular arrangement of the surrounding assembles [23], the explanation of which is beyond the scope of this paper.

## 4. Conclusions

We were able to map the magnetic configuration of ensembles of Fe_3_O_4_ nanocubes, approximately 40 nm in size, by means of electron holography. The self-assembly of the nanocubes in the form of chains was confirmed to be driven both by the shapes of the blocks and by the dipole–dipole interaction. Furthermore, a very good agreement between simulated and experimental phase shift maps is obtained.

In this regard, our former work unambiguously demonstrate the important role of chain alignment on the area of hysteresis loop (and therefore of the SAR) [24]. Consequently, disorientation of the assembly and deviations from the homogeneous flux distribution (as the ones reported in Figure 6) would lead to a considerable decrease in the heating efficiency and can most probably explain the smaller SAR values for the 40 nm sample compared to the 20 nm case [11].

It is our view that our findings contribute to the knowledge on the complexity of the magnetic structure in applications as diverse as non-volatile storage devices and cancer therapies, which calls for further studies.
